# A Phytoprostane from *Gracilaria longissima* Increases Platelet Activation, Platelet Adhesion to Leukocytes and Endothelial Cell Migration by Potential Binding to EP3 Prostaglandin Receptor

**DOI:** 10.3390/ijms24032730

**Published:** 2023-02-01

**Authors:** Silvia Montoro-García, Sara Martínez-Sánchez, Miguel Carmena-Bargueño, Horacio Pérez-Sánchez, María Campillo, Camille Oger, Jean-Marie Galano, Thierry Durand, Ángel Gil-Izquierdo, José Antonio Gabaldón

**Affiliations:** 1 Izpisua Lab, HiTech, Health Sciences Department, Universidad Católica de Murcia (UCAM), Campus de los Jerónimos 135, 30107 Guadalupe, Spain; 2 Structural Bioinformatics and High-Performance Computing Research Group (BIO-HPC), Computer Engineering Department, Universidad Católica de Murcia (UCAM), Campus de los Jerónimos 135, 30107 Guadalupe, Spain; 3 Institut des Biomolécules Max Mousseron (IBMM), UMR 5247-CNRS, Faculty of Pharmacy, University of Montpellier-ENSCM, 34093 Montpellier, France; 4 Research Group on Quality, Safety and Bioactivity of Plant Foods, Department of Food Science and Technology, CEBAS-CSIC, University Campus of Espinardo, Edif. 25, 30100 Espinardo, Spain; 5 Molecular Recognition and Encapsulation Research Group (REM), Health Sciences Department, Universidad Católica de Murcia (UCAM), Campus de los Jerónimos 135, 30107 Guadalupe, Spain

**Keywords:** phytoprostanes, prostaglandins, platelets, EP3 receptor, migration, inflammation

## Abstract

Plant phytoprostanes (PhytoPs) are lipid oxidative stress mediators that share structural similarities with mammal prostaglandins (PGs). They have been demonstrated to modulate inflammatory processes mediated by prostaglandins. The present study aims to test the effects of the most abundant oxylipin from *Gracilaria longissima*, ent-9-D1t-Phytoprostane (9-D1t-PhytoP), on platelet activation and vascular cells as well as clarify possible interactions with platelets and the endothelial EP3 receptor Platelet and monocyte activation was assessed by flow cytometry in the presence of purified 9-D1t-PhytoP. Cell migration was studied using the human Ea.hy926 cell line by performing a scratch wound healing assay. The RNA expression of inflammatory markers was evaluated by RT-PCR under inflammatory conditions. Blind docking consensus was applied to the study of the interactions of selected ligands against the EP3 receptor protein. The 9D1t-PhytoP exerts several pharmacological effects; these include prothrombotic and wound-healing properties. In endothelial cells, 9D1t-PhytP mimics the migration stimulus of PGE_2_. Computational analysis revealed that 9D1t-PhytP forms a stable complex with the hydrophobic pocket of the EP3 receptor by interaction with the same residues as misoprostol and prostaglandin E_2_ (PGE_2_), thus supporting its potential as an EP3 agonist. The potential to form procoagulant platelets and the higher endothelial migration rate of the 9-D1t-PhytoP, together with its capability to interact with PGE_2_ main target receptor in platelets suggest herein that this oxylipin could be a strong candidate for pharmaceutical research from a multitarget perspective.

## 1. Introduction

The discovery of plant oxylipins in the 1990s marked a breakthrough in oxidized free fatty acids. These molecules, similar to human prostaglandins (PGs, mammal oxylipins) [1], are generated through the oxidation of polyunsaturated fatty acids such as α-linolenic acid, without the participation of the enzyme cyclooxygenase (COX), as occurs in humans [2,3,4]. Oxidative stress in plants can lead to the dysregulation of lipid metabolism and the release of multiple lipid mediators, such as phytoprostanes (PhytoPs, plant isoprostanes) and phytofurans (PhytoFs), participating in the redox balance in different crops [2,5,6,7]. Recently, PhytoPs have been extensively characterized in a wide diversity of plant-based foods, including nuts, hazelnuts, almonds, vegetable oils, cereals, olives, wine, peas, rice, several tropical fruits, cocoa, macroalgae, chocolate, pistachios, dates, and especially in legumes, at different concentrations [8,9,10]. Moreover, oxylipins extracted from the red macroalgae *Gracilaria longissima* have been shown to modulate pro-inflammatory responses associated with endothelial dysfunction [11]. In another recent paper, distinct purified oxylipins were able to prevent the up-regulation of PGs metabolites induced by lipopolysaccharide in THP1 cells, protecting against inflammatory disorders [12]. Therefore, oxylipins have a myriad of functions that are still being elucidated.

In humans, prostanoids are bioactive ligands characteristic of distinct cell types (endothelium, platelets, smooth muscle, neurons, among others) and physiological states. Originating from arachidonic acid (AA) and PG endoperoxides, the most relevant prostanoids for platelet and vascular function are thromboxane A_2_ (TxA_2_), prostaglandin D_2_ (PGD_2_), prostaglandin E_2_ (PGE_2_), and prostacyclin (PGI_2_). These prostanoids exhibit subtle differences in their chemical structure and can bind a wide diversity of prostanoid receptors on the surface of platelets, herein sustaining homeostasis and playing an important role in pathogenic mechanisms, including the inflammatory response, platelet aggregation, and vascular tone regulation [13]. More recently, PGE_2_ has been found to present four EP receptor subtypes (EP1, EP2, EP3, and EP4) that trigger G-protein-coupled signaling pathways [14]. Among them, EP3 and EP4 receptors are much more prominent in human platelets and endothelium and appear to be valuable drug targets [15,16]. Consistent with these findings, a recent study showed PGE_2_ can bind either a pro-thrombotic (EP3) or anti-thrombotic (EP4) receptor on the surface of platelets [17].

Additionally, PhytoPs are geometric isomers of human PGs, more concretely of PGE_2_, where the PhytoPs side chains display a *cys* configuration, compared to the *trans* of PGs [1]. That is why, biologically, PhytoPs could potentially exert their effect through the activation of prostanoid receptors and further intracellular signaling. However, evidence supporting a role for PhytoPs in the regulation of platelets and prostanoids sensitive cells is lacking. Furthermore, to identify the underlying mechanism regulated by a PhytoP of interest, its corresponding receptor must be elucidated. It is then understandable why the in vitro and in silico characterization of these purified compounds individually could improve our understanding of their biological implications in health and disease. The present study aims to test the effects of ent-9-D1t on platelet activation markers and human vascular cells, as well as to clarify with docking simulations possible interactions with the EP3 receptor.

## 2. Results

### 2.1. Individual PhytoPs and PhytoFs Composition in Gracilaria Longissima Extract

The analysis of the individual and total oxylipins was performed by UHPLC-ESI-QqQ-MS/MS. The most abundant species of oxylipin in the extract was the compound 9-D1t-PhytoP (FP-2), being 29-fold higher than the second most abundant PhytoPs and PhytoFs (Table 1).

### 2.2. PhytoPs Extract Stimulates Platelet Activation and Aggregation

**Figure 1:**
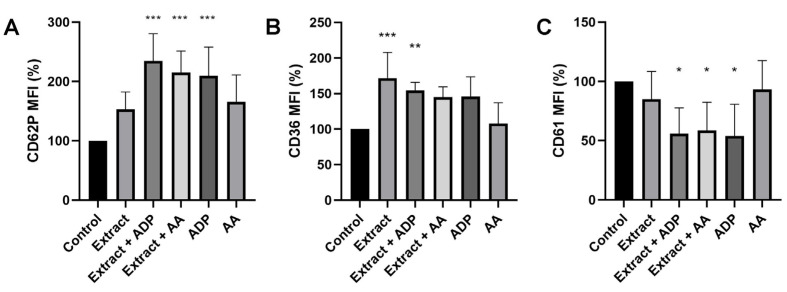
Effects of the *Gracilaria longissimi* extract rich in 9-D1t- on the expression of platelet activation markers. (**A**) CD62P (P-selectin) MFI. (**B**) CD36 (glycoprotein IV) MFI. (**C**) CD61 (glycoprotein IIIa) MFI. The final concentrations in citrated blood were: 129 nM extract, 20 μmol/L ADP, and 3 μg/mL AA. MFI: Mean fluorescence intensity. The bars show mean and standard deviation (n = 9 different blood donors). * *p* < 0.05; ** *p* < 0.01; *** *p* < 0.001 vs. control condition (PBS).

We first tested the effects of a 9-D1t-PhytoP-rich extract on platelet activation markers in whole blood. The preincubation with the extract alone was only able to upregulate glycoprotein IV (CD36), an atherosclerotic stress marker (*p* = 0.0009) (Figure 1B). However, the extract did not impair the CD62P and CD36 expression induced by ADP and AA (both *p* < 0.001) (Figure 1A,B). A good correlation was observed between CD62P and CD36 mean fluorescence intensity (MFI) for all the conditions. Finally, platelet aggregation (decrease in CD61 MFI) was induced by ADP in whole blood (*p* < 0.05), but also pretreated with extract/ADP and extract/AA, showing a synergistic effect with AA (all *p* < 0.05, Figure 1C). Arachidonic acid alone was not able to stimulate platelet aggregation in a significant way (Figure 1C). These results indicate that *Gracilaria sp*. Extract alone induces platelet activation, but it was not sufficient for an aggregation response.

### 2.3. 9-D1t-PhytoP Affects Platelet Aggregation through EP Receptors

**Figure 2:**
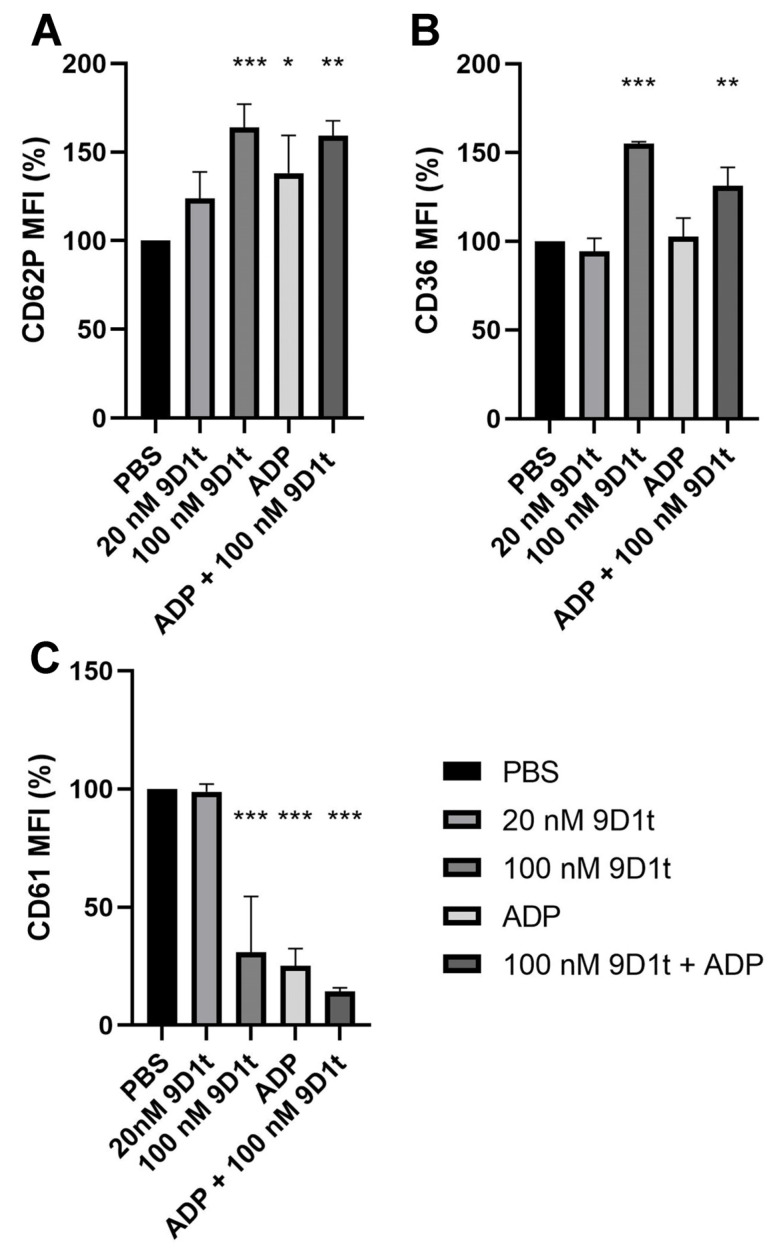
Effects of 9-D1t-PhytoP on the expression of platelet activation markers. (**A**) CD62P (P-selectin) expression. (**B**) CD36 (glycoprotein IV) expression. (**C**) CD61 (glycoprotein IIIa) expression. The final concentrations in citrated whole blood were: 20-100 nM 9-D1t-PhytoP and 20 μmol/L ADP. MFI: mean fluorescence intensity. The bars show mean and standard deviation (n = 5 different blood donors). * *p* < 0.05; ** *p* < 0.01, *** *p* < 0.001 vs. control condition (PBS).

We then tested the hypothesis that 9-D1t-PhytoP treatment modulates platelet activation induced by ADP. Citrated whole blood (n = 5 blood donors) was treated with 20 and 100 nM purified 9-D1t-PhytoP for 10 min before ADP stimulation. In Figure 2A,B, the ability of 100 nM 9-D1t-PhytoP to increase P-selectin and CD36 expression on single platelets was significant (both *p* < 0.001). Treatment with 20 nM of 9-D1t-PhytoP alone did not trigger platelet aggregation (*p* = 0.723) but a higher concentration (100 nM) did (*p* < 0.001, Figure 2C). 9-D1t-PhytoP was also able to enhance the extent of ADP-induced platelet aggregation (*p* < 0.001), which was consistent with previous data with *Gracilaria longissima* extract rich in 9-D1t-PhytoP.

As platelets express mainly two receptors for prostanoids (EP3 and EP4 receptors), the effects of 100 nM 9-D1t-PhytoP on platelet activation in whole blood, induced by ADP as an agonist, were determined in the presence of selective EP3 and EP4 receptor ligands (Figure S1). At this high ADP concentration (20 µM), we did not observe any effect of 40 nM PGE_2_ preincubation on platelet aggregation (CD61) or CD62P expression (Figure S1A–C), even in the presence of 100 nM 9-D1t-PhytoP, although CD36 expression tended to increase (*p* = 0.056, Figure S1B). The combination of ligands and 100 nM 9-D1t-PhytoP slightly displayed a synergistic effect in the presence of 200 nM Sulprostone for CD36 (*p* = 0.051). Nonetheless, the stimulation with ADP did not seem adequate for the 9-D1t-PhytoP characterization. Therefore, the same experiment was performed without ADP as an agonist.

**Figure 3:**
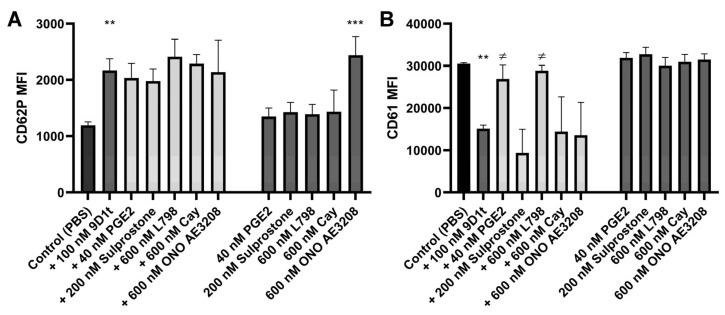
Effects of 100 nM 9-D1t-PhytoP alone or in combination with distinct EP receptor ligands on the expression of platelet activation markers. (**A**) CD62P (P-selectin) expression. (**B**) CD61 (glycoprotein IIIa) expression. Samples of whole blood were stimulated for 10 min and analyzed by flow cytometry. MFI: Mean fluorescence intensity. The bars show mean and standard deviation (n = 5 different blood donors). ** *p* < 0.01, *** *p* < 0.001 vs. control condition (PBS); and ≠ *p* < 0.05 vs. 9-D1t-PhytoP.

In the absence of an ADP agonist (Figure 3), the distinct modulators alone were unable to significantly stimulate platelet activation and aggregation (all *p* > 0.05). Only ONO AE3-208 (EP4 antagonist) displayed a prothrombotic profile (Figure 3A,B) (*p* = 0.0007 for CD62P), in a similar pattern as 9-D1t-PhytoP (*p* = 0.018). Moreover, platelet aggregation (CD61 decrease) induced by 9-D1t-PhytoP (CD61, *p* = 0.0146), was blocked by the EP3 ligands, PGE_2_ (agonist, *p* = 0.048) and L798-106 (antagonist, *p* = 0.015), denoting thus a possible competition for the same receptor, but not by Sulprostone (*p* = 0.83). No changes appeared in these platelet markers when 9-D1t-PhytoP and EP4 modulators were incubated together, compared to 9-D1t-PhytoP alone (all *p* > 0.05).

**Figure 4:**
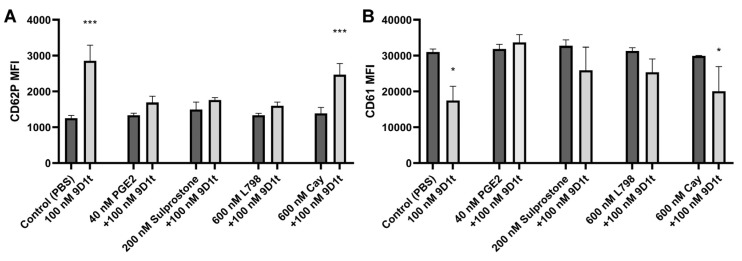
The EP4 agonist Cay10598 inhibits the effect of 9-D1t-PhytoP. (**A**) CD62P (P-selectin) expression. (**B**) CD61 (glycoprotein IIIa) expression. Samples of whole blood were preincubated with EP3/EP4 ligands (black) for 10 min plus 100 nM 9-D1t-PhytoP (gray) for additional 10 min at 37 °C and analyzed by flow cytometry (n = 5). MFI: mean fluorescence intensity. * *p* < 0.05; *** *p* < 0.001 vs. control condition (PBS).

Next, we examined the effects of 9-D1t-PhytoP on platelet activation markers by blocking the EP3/EP4 receptors first. Accordingly, we found a reduction in activation/aggregation in the presence of EP3 ligands, denoting thus the contribution of 9-D1t-PhytoP to the aggregation via the EP3 receptor (Figure 4, all *p* > 0.05), whereas in the presence of Cay10598, treatment with 100 nM 9-D1t-PhytoP stimulated activation and aggregation (CD62P *p* < 0.001 and CD61 *p* = 0.358, respectively).

### 2.4. 9-D1t-PhytoP Alone Does Not Increase Platelet Adhesion to Leukocytes

There is evidence that the overall effects of prostanoids on human platelet function are the consequence of a balance between stimulatory effects exerted at the EP3 receptor and inhibitory effects acting at the EP4 receptor. For that purpose, we tested the effects of ligands of these two receptors in combination with purified 9-D1t-PhytoP and examined the platelet adhesion to leukocytes by FCM (Figure S2, Q2: CD14+CD42b+).

**Figure 5:**
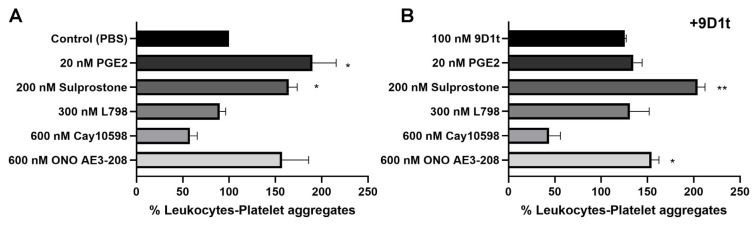
9-D1t-PhytoP effect on the formation of platelet–leukocyte in fresh EDTA blood. (**A**) Relative counts in the presence of EP3/EP4 ligands. (**B**) Relative counts in the presence of EP3/EP4 ligands plus 100 nM 9D1t. Values are normalized to control (PBS). Values are mean ± SD (n = 3 with different blood donors). Stars denote significant differences as compared to samples without activation (PBS, control) */** *p* < 0.05/0.01, * *p* value derived from 2-tailed paired *t*-test.

As shown in Figure 5, treatment with the EP3 agonists (PGE_2_ and Sulprostone) increased platelet binding, similar to the EP4 antagonist (ONO AE3-208) (all *p* < 0.05). Treatment with 9-D1t-PhytoP alone, or in combination with 20 nM PGE_2_, did not trigger platelet adhesion to leukocytes (123.98% and 135.34%, respectively, *p* > 0.05), although a synergistic effect was found when fresh blood was treated with Sulprostone in addition to 9-D1t-PhytoP (204.18%, *p* < 0.01). Interestingly, the effect of the EP3 antagonist L798,106 was slightly counteracted by the addition of 9-D1t-PhytoP (93.19% vs. 121.30%), denoting a potential competition for the same EP receptor (*p* > 0.05).

To confirm that the effect of 9-D1t-PhytoP was mediated via EP3 receptor activation, fresh blood was also treated with the EP4 ligands (ONO AE3-208 and Cay10598) together with 100 nM 9-D1t-PhytoP. The presence of 9-D1t-PhytoP did not influence the inhibitory effect of the EP4 agonist Cay10598. Conversely, 9-D1t-PhytoP had no effect on the ONO AE3-208-induced platelet adhesion to leukocytes (Figure 5B), thus suggesting that the modulatory effect of 9-D1t-PhytoP was mediated predominantly by the EP3 receptor.

### 2.5. 9-D1t-PhytoP Does Not Compromise Endothelial Cell Viability

Before testing the capacity of purified 9-D1t-PhytoP to modulate gene expression and migration on endothelial cells, the cytotoxic effect on the line Ea.hy926, a well-established model of endothelial cell-based studies, was assessed. Treatment with 9-D1t-PhytoP was not found to affect the viability or proliferation up to 1 µg/mL for the first 48 h. However, at higher concentrations (1 µg/mL) for 6 days, the viability was significantly reduced (*p* < 0.05) (Figure S3).

### 2.6. 9-D1t-PhytoP Bioactivity over the Endothelium

#### 2.6.1. 9-D1t-PhytoP Contributes to In Vitro Migration

**Figure 6:**
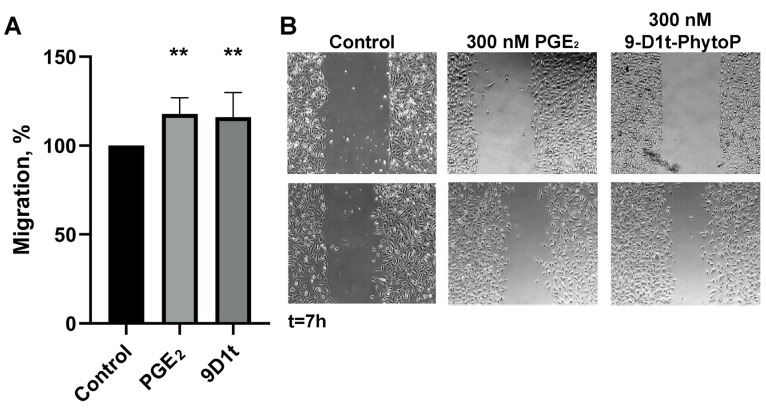
Migration (wound closure) in human endothelial Ea.hy926 cells in the presence of 300 nM PGE_2_ and 9-D1t-PhytoP, respectively. (**A**) Percentage of migrated area following the wound healing assay (n = 3). (**B**) Representative images of the wound healing assay at t = 0 and t = 7 h. Statistic differences compared with control ** *p* < 0.01.

PGE_2_ is known to stimulate migration and angiogenesis in endothelial cells. As shown in Figure 6, 300 nM 9-D1t-PhytoP significantly increased migration in a similar manner as PGE_2_ (*p* = 0.004, respectively).

#### 2.6.2. 9-D1t-PhytoP Induces an Endothelial Dysfunctional Status

Gene expression of NOS3, COX-2 (PGST), ICAM-1, and IL-6 were measured after 9-D1t-PhytoP and TNF-α treatments (Figure S4). 9-D1t-PhytoP alone did not produce significant changes in NOS3, ICAM-1, nor PGST expression in endothelial cells. However, TNF-α treatment was able to decrease NOS3 expression (*p* = 0.012, and increase ICAM-1 (*p* = 0.0001), PGST (*p* = 0.0002), and IL-6 expression (*p* = 0.0262). Pretreatment with 300 nM 9-D1t-PhytoP and TNF-α showed a synergistic effect on NOS3 and ICAM-1 expression (*p* = 0.0087).

### 2.7. Computational Methods for Discovering the Biological Receptors of 9-D1t-PhytoP

#### 2.7.1. 9-D1t-PhytoP Binds to the Hydrophobic Pocket of EP3

**Figure 7:**
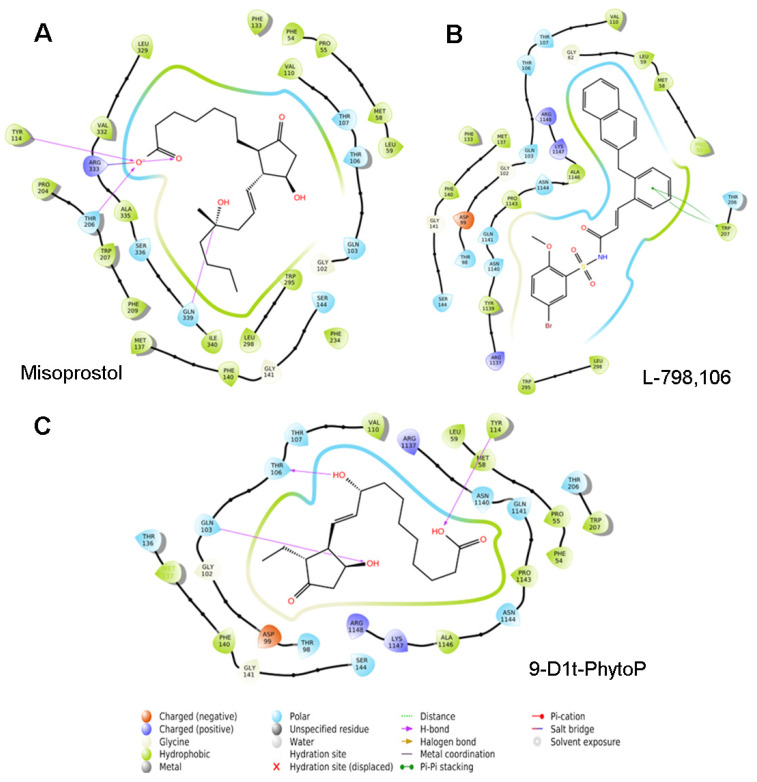
Two-dimensional protein–ligand interactions diagram generated between EP3 receptor (PDB: 6M9T) and (**A**) Misoprostol (EP3 agonist); (**B**) L-798,106 (EP3 antagonist); and (**C**) 9-D1t-PhytoP ligands, using the Ligand Interaction script in Maestro (Schrödinger Inc., www.schrodinger.com, accessed on 14 May 2022). It outlines a cavity consisting of several proximate hydrophobic and hydrophilic residues (Thr 106 and 107, shown in blue circles) where 9-D1t-PhytoP potentially binds. A hydrogen bond between the ligand and Gln 103 is shown by an arrow.

We then performed blind docking (BD) approaches with the EP3 receptor (PDBs: 6M9T). Our model system compared the clusters of their well-characterized agonists (misoprostol and PGE_2_) and antagonists (L-798,106). Figure 7C shows the potential binding of 9-D1t-PhytoP to the hydrophobic pocket of EP3, in a similar way to Misoprostol (synthetic prostanoid, EP3 agonist) (energy binding e-11, Figure S5).

#### 2.7.2. 9-D1t-PhytoP Shares Hydrophobic Interactions with EP3 Agonists

**Figure 8:**
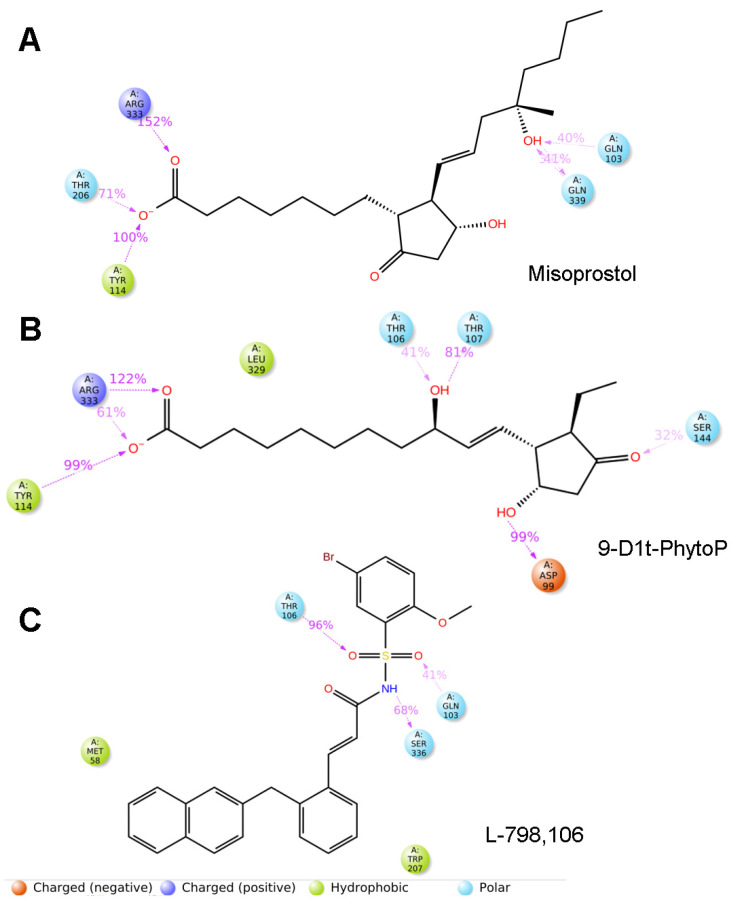
Two-dimensional protein–ligand interactions of the molecular dynamics diagrams generated between EP3 receptor (PDB: 6M9T) and (**A**) Misoprostol (EP3 agonist), (**B**) 9-D1t-PhytoP, and (**C**) L-798,106 (EP3 antagonist) using the Simulation Interactions Diagram script in Maestro (Schrödinger Inc., www.schrodinger.com, accessed on 20 December 2022).

In order to give more information about the molecular recognition of 9-D1t-PhytoP to EP3, 100 ns MD simulations were rendered, and the binding of the PhytoP to the hydrophobic cavity remains stable with the top averaging interactions shown in Figure 8. PGE_2_, Misoprostol, and 9-D1t-PhytoP share hydrophobic interactions with Arg333, Tyr114, and Thr206, whereas the EP3 antagonist, L-798,106, does not (Figure 8 and Figure S6). On the contrary, L-798,106 does not reach far down into the binding pocket, which could lead to a non-activating conformation.

## 3. Discussion

In order to understand the modulating vascular and platelet activity of PhytoPs as bioactive ingredients from plant foods, it is necessary to delve into the specific function of the different EP receptor subtypes. Thus, the current study aimed at characterizing (1) the effect of purified 9-D1t-PhytoP on platelet activation and aggregation, (2) the effects of 9-D1t-PhytoP on vascular cells such as monocytes in the presence of EP selective modulators, (3) the migration and inflammatory protein expression changes in endothelial cells, and (4) the mechanistic binding to the EP3 receptor. The results of these studies suggest that 9-D1t-PhytoP acts as a fine tuner of platelet function primarily through its potential interaction with EP3, allowing diverse pharmacological strategies useful in cardiovascular treatment and prevention.

The high structural homology of PhytoPs to human prostanoids such as PGE_2_ has attracted tremendous appreciation in recent years, especially by unraveling the potential biological activities that they could exert in human vascular cells and platelets. PGE_2_ can bind at least four structurally different receptor subtypes (EP1–4), resulting in diverse and often opposite final biological responses [18]. Moreover, a “dual” effect of PGE_2_ on platelet response (i.e., activatory at nanomolar concentrations and inhibitory at micromolar concentrations) was repeatedly observed in early studies [15,19]. Much research on the use of prostanoids such as PGE_2_ and its synthetic analogs (Sulprostone, Misoprostol) over their selective EP3 receptor subtype has been performed [20,21]. Nonetheless, as previously reported, such agonists mimic the effects of low concentrations of PGE_2_ on platelets induced by ADP, but they do not enhance platelet aggregation without agonist stimulation [22,23]. Further, multiple mammal prostanoids (i.e., enhanced thromboxane production) are synthesized in response to distinct agonists making it difficult to attribute the final cellular response to any one particular compound (PhytoPs, AA, thrombin, etc.). As platelet activation mediated by ADP is independent of the lipoxygenase- and cyclooxygenase-dependent metabolites of arachidonic acid, and in order to avoid confounding crosstalk, we selected ADP as the agonist [24].

As the most abundant oxylipin found in *Gracilaria longissima* extract was the 9-D1t-PhytoP, the study aimed at evaluating its particular bioactivity over vascular elements, as well as deciphering the potential receptors in humans. For this, our previous work studied the effect of *Gracilaria longissima* extract rich in 9-D1t-PhytoP in endothelial cells and found that it did not provide significant mitigation of the pro-inflammatory changes induced by TNF-α but has not been tested in human platelets yet [11]. In contrast, in another study, the ent-9-D1t-PhytoP reduced pro-inflammatory markers in monocytes to values found in the untreated control [12]. The functions of only a few oxylipins have been characterized in platelets, and those that have been characterized still remain controversial [25]. Herein, we focused now on the potential that the 9-D1t-PhytoP-EP3 might display in thrombus formation, by regulating platelet activation and aggregation. In the current experiments, we addressed the possibility that the effect of the 9-D1t-PhytoP was mediated by PG receptors mainly through the EP3 receptor, Moreover, we measured the effect of 9-D1t-PhytoP in platelets pre-treated with an EP3 antagonist (L798,106) and an EP4 agonist (Cay10598) on the expression of P-selectin (secreted from α-granules) and CD36 with and without ADP stimulation. The results of these experiments point out that 9-D1t-PhytoP has a stimulatory effect on platelet function via the EP3 receptor, under non-aggregating conditions. It is important to notice that the opposing anti-aggregatory action of PGE_2_ for 9-D1t-PhytoP through the IP receptor has not been tested. Moreover, the high potency of 9-D1t-PhytoP as a potentiator implies that an EP2 receptor is unlikely to be involved [16]. EP2 receptors are associated with the relaxant actions of PGE_2_ on vascular and respiratory smooth muscles, and Sulprostone is virtually inactive in these systems [26]. Furthermore, it is likely that the EP3 rather than the epinephrine subtype mediates the potentiating effect, as 9-D1t-PhytoP is more potent than PGE_2_ and Sulprostone in the current conditions.

Extensive FCM approaches have considerably extended our knowledge of platelet–leukocyte interactions and their implications on health and disease [27,28]. We now tested the effect of 9-D1t-PhytoP on the capacity of platelets to form platelet–leukocytes aggregates. The platelet binding to other vascular cells in the presence of 9-D1t-PhytoP provided an amplification of the thrombotic state, which could become a therapeutic target in various diseases. Furthermore, leukocyte recruitment plays a significant role in the early phase of inflammation and endothelial dysfunction. It is well established that PGE_2_ induces endothelium migration [29], but we also confirmed that the high dose of 9-D1t-PhytoP contributes to in vitro migration as well. Finally, the activation of endothelium promoted IL-6 overexpression (Figure S4D), confirming the data that high doses of the EP ligand are able to induce a dysfunctional status. Moreover, one has to keep in mind that arterial and venous endothelial cell lines have been used in the literature with controversial differences in the release of vasoactive substances such as PGs or TxA_2_ [30,31]. We assessed the effects of 9-D1t-PhytoP in arterial endothelial cells (Ea.hy926 cell line) in order to give continuity to our previous studies [11,32].

In order to understand the precise binding mode of the 9-D1t-PhytoP and its potential EP3 receptor, a combination of docking and MD was performed. The EP3 receptor presents dual signaling that requires extensive structural studies [33,34]. The residues that made high-energy contributions to the final poses of EP3-misoprostol, EP3-9-D1t-PhytoP, and EP3-PGE_2_ were R333, T206, and Y114, providing a disparate binding model to EP3-L-798,106. Indeed, recent crystallographic studies have reported molecular details of such interactions; the tested agonists tend to adopt an L-shaped conformation in the EP3 binding site, which is important for receptor activation, and 9-D1t-PhytoP perfectly overlaps with it [33,34].

These results suggest that PhytoPs may have prothrombotic and pro-invasive effects, in addition to their known immunomodulatory abilities [12], in a similar manner as mammal PGE_2_. The originality of the current work lies in the combination of ex vivo platelet activation together with classical endothelial in vitro approaches. Although current results are very promising, further studies are needed to establish the EP receptors’ affinity of this and other PhytoPs and perhaps a dual role with distinct prostanoid receptors. It is also important to implement in vivo studies of the extracts with the aim of confirming a clinical effect.

## 4. Materials and Methods

### 4.1. Chemicals and Reagents

The PhytoPs: 9-F1t-PhytoP; ent-16-F1t-PhytoP, ent-16-epi-16-F1t-PhytoP; 9-epi-9-F1t-PhytoP; 9-D1t-PhytoP; 9-epi-9-D1t-PhytoP; 16-B1-PhytoP; and 9-L1-PhytoP; as well as the PhytoFs: ent-16(RS)-9-epi-ST-Δ14-10-PhytoF; ent-9(RS)-12-epi-ST-Δ10-13-PhytoF; and ent-16(RS)-13-epi-ST-Δ14-9-PhytoF were synthesized according to already published procedures [35] and provided by the *Institut des Biomolecules Max Mousseron* (IBMM) (Montpellier, France). PGE_2_ and other agonists of EP3/EP4 receptors (Sulprostone and Cay10598, respectively), as well as antagonists (L-798,106 and ONO AE3-208, respectively), were purchased from Cayman Chemicals (Ann Arbor, MI, USA).

### 4.2. Analytical Extract Rich in Oxylipins

The oxylipins from *Gracilaria longissima* were extracted and characterized as previously published [8,11]. Briefly, 1 g of powdered algae was mixed with 5 mL of methanolic butylated hydroxyanisole (BHA) (99.9: 0.1, *v*/*w*), and the extracts were cleaned up by SPE. The PhytoP and PhytoF extracts were prepared at room temperature (RT). After SPE, the extracts were then reconstituted in 200 μL of MeOH/MilliQ-water (50/50, *v*/*v*), filtered through a 0.45 μm filter (Millipore, MA, USA), and immediately analyzed by UHPLC-ESI-QqQ-MS/MS (Agilent Technologies, Waldbronn, Germany).

### 4.3. In Vitro Assessment of Platelet Activation and Polymorphonuclear Leukocytes (PMNs)-Platelet Aggregates

Nine healthy participants (without any treatment) were recruited among the staff of the Catholic University of Murcia (UCAM). Fresh blood samples were collected in commercial 2 mL sodium citrate (32%) and EDTA Vacutainer tubes using a 20-gauge needle (the first 2 mL was discarded). The blood extractions were performed in accordance with the Helsinki declaration and approved by the UCAM Research Ethics Committee. All participants provided written informed consent.

Saline dilutions of 0.2 µg of characterized oxylipins (Table 1) and agonists were freshly prepared and added to 40 µL of fresh citrated blood (rending 129 nM) for 10 min, followed by platelet activation marker analysis by flow cytometry (FCM). The platelet subpopulations (normal platelets and platelet aggregates (PA)) in whole blood were distinguished from other cells based on the presence of constitutive platelets’ membrane receptor CD42b (glycoprotein Ib) and CD61 (glycoprotein IIIa, fibrinogen receptor activation, and the formation of platelet aggregates). The surface expression of platelet membrane activation markers such as CD62P (P-selectin) and CD36 (glycoprotein IV) was then assessed as already published [28,36]. After incubation with extract and EP receptor ligands for 10 min, the stimulated whole blood (5 µL) was labeled with mouse–anti-human CD61-fluorescein isothiocyanate (FITC), mouse–anti-human CD62P-BV786, mouse–anti-human CD42b-allophycocyanin (APC), and CD36-phycoerythrin (PE) (all from BD Biosciences, Oxford, UK) for 15 min, diluted with 1 mL of filtered PBS and then analyzed in a FACS Fortessa flow cytometer (BD, Becton Dickinson, Oxford, UK). Positive controls of platelet activation were also assessed after the incubation of the whole blood for 2 min with adenosine-5′-diphosphate (ADP, 0.02 mmol/L; Biodata Corporation, Horsham, PA, USA) and arachidonic acid (AA, 250 µg/mL; Biodata Corporation, Horsham, PA, USA), giving a monophasic, complete, and irreversible aggregation [37].

Mouse antihuman monoclonal fluorochrome-conjugated antibody anti-CD14-BUV395 and CD42b-APC were mixed with 50 μL of fresh EDTA anticoagulated whole blood in TruCount tubes (all from BD) containing a strictly defined number of fluorescent count beads. After incubation for 15 min, red blood cells were lysed by 450 μL of lysing solution (BD, Oxford, UK) for 15 min, followed by dilution in 1 mL of PBS and immediate flow cytometric analysis. Monocytes and other polymorphonuclear leukocytes (PMNs) were selected by gating strategies based on forward and side scatter to select monocytes and side scatter versus CD14 expression to exclude granulocytes (some lymphocytes and neutrophiles might be included in this gate too) (Figure S2). Absolute counts of CD14+ leukocytes (in cells per microliter) were obtained by calculating the number of gated events proportional to the number of the count beads according to the manufacturer’s recommendations. CD14+ leukocyte–platelet aggregates were defined as events positive to CD14 and the platelet marker CD42b [27]. The number of events collected was 10,000 for the CD14+ leukocyte gate subset. Isotype controls for all the FCM assays were performed with different monoclonal anti-IgG1 (BD Biosciences).

Citrated and EDTA anticoagulated blood was incubated with ligands of platelet EP3 and EP4 receptors for 10 min at room temperature (RT). In total, 200 nM Sulprostone (agonist) and 600 nM L798,106 (antagonist) were used to stimulate the EP3 receptor. Whilst 600 nM Cay10598 (agonist) and ONO AE3-208 (antagonist) were used to stimulate the EP4 receptor, respectively, 20–40 nM PGE_2_ was also used in the same conditions. Whole blood was incubated with 20–100 nM purified 9-D1t-PhytoP plus the above ligands and then processed by FCM.

### 4.4. Cell Line and Culture Conditions

Endothelial Ea.hy 926 (ATCC^®^CRL2922) human cell line was obtained from the American Type Culture Collection (ATCC, Rockville, MD, USA). Cells were grown in DMEM containing 4.5 g L^−1^ glucose, supplemented with GlutaMAX™, 10% fetal bovine serum (FBS), and 1% non-essential amino acids at 37 °C in a humidified atmosphere containing 5% CO_2_. The passage number of the cells used in this study was between 3–8 for the Ea.hy926 cell line.

### 4.5. Cell Viability Assay

The toxicity of 9-D1t-PhytoP (up to 1 µM, 0.01% DMSO) dissolved in complete medium was tested in Ea.hy 926 cells. For this, exponentially growing cells were seeded into a 96-well plate at a density of 10^4^ cells/well. After the time of incubation (48 h and 6 days) with growing concentrations of 9-D1t-PhytoP, the cells were incubated with AlamarBlue solution at 10% concentration (ThermoFisher, Massachusetts, USA) for 4 h at 37 °C. The absorbance of the media was measured in a microplate reader (Bio-Tek Synergy HT, Winooski, VT, USA) at 570 nm and 600 nm of reference. Cell viability was calculated as the average optic density (OD) of the wells/average OD of control wells and expressed as a percentage (%).

### 4.6. Cell Migration Assay

Cell migration was studied using the Ea.hy926 cell line by performing a scratch wound healing assay in standard medium supplemented with 5% FBS. Typically, 30,000 cells were plated in low 35 mm dishes with culture inserts following the manufacturer’s instructions (Ibidi, Martinsried, Germany). After appropriate cell attachment and monolayer formation (around 24 h), inserts were removed with sterile forceps to create a wound field of approximately 500 µm. Detached cells were gently removed with DPBS before treatment. Confluent cells were incubated in one of the following treatments: control (0.01% DMSO), 300 nM PGE_2_, or 300 nM 9-D1t-PhytoP. The cells were then placed in a cell culture incubator, and they were allowed to migrate. At 0 and 7 h (linear growth phase) [38,39], 10 fields of the injury area were photographed with an inverted phase contrast microscope using 10× magnification. For each time point, the area uncovered by cells was determined by Image J software (National Institute of Health, Bethesda, MD, USA). Each treatment was performed in triplicate.

The migration speed of the wound closure was given as the percentage of the recovered area at each time point, relative to the initially covered area (t_0_). The velocity of wound closure (%/h) was calculated according to the following equation and slopes are expressed as percentages relative to control conditions: Slope (%area/h) = (% covered area/t_x_) − [(% covered area t_0_) * (t_x_ − t_0_)].

### 4.7. Biological Activity in an Endothelial Dysfunction Model

In order to study the effects that 9-D1t-PhytoP produced on the EA.hy926 phenotype, cells were seeded in 24-well plates at 8 × 10^4^ cells/well in DMEM supplemented with 5% FBS and treated with 100 nM 9-D1t-PhytoP (per triplicate) for 16 h, as previously published [11]. Then, 20 ng/mL TNF-α was added for an additional 6 h of incubation time.

Total RNA was extracted from EA.hy926 cells using 300 µL Trisure (Bioline, Taunton, MA, USA) reagent and Direct-Zol RNA MiniPrep (Zymo Research Irvine, Irvine, CA, USA) according to the manufacturer’s protocol. Total RNA was reverse-transcribed into complementary DNA by using a Sensifast cDNA™ Synthesis kit (Bioline, Taunton, MA, USA). The mRNA levels of the target genes were quantified by RT-PCR using a SensiFAST SYBER Hi-ROX Kit (Bioline, Taunton, MA, USA) in a StepOnePlus Real-Time PCR System (Applied Biosystems, Foster City, CA, USA). Briefly, 5 µL of 1:5 diluted cDNA was added to the qPCR reaction containing 10 µL 2X SensiFAST Mix and 400 nM of each primer in a total volume of 20 µL.

Specific and validated primers for distinct human genes such as actin, intercellular adhesion marker-1 (ICAM-1), vascular cell adhesion marker-1 (VCAM-1), endothelial nitric oxide synthase (NOS3), interleukin-6 (IL-6), and COX-2 (PGST) were purchased (all from Sigma-Aldrich Chemical Co., Saint Louis, MO, USA). The relative mRNA expression of the genes of interest was represented by: 2ˆ(-DDCT) = [CT (gene of interest) − CT (Actin)]test − [CT (gene interest) − CT (Actin)] control.

The relative quantification of gene expression was evaluated by the comparative fold change 2ˆDDCT method [40]. An average value of each target gene after actin normalization at the time point showing the highest expression was used as a calibrator to determine the relative levels in the rest of the experimental conditions. All the experiments and qPCR reactions were performed in triplicate.

### 4.8. Blind Docking

Blind docking (BD) consensus was applied to the study of the interactions of selected ligands against the EP3 receptor protein. The BD method allows to calculate the most probable interaction hotspots for a given ligand across the selected protein surface [41].

Regarding input file preparation, the crystallized structure of the EP3 receptor was obtained from the PDB database with the code 6M9T (https://www.rcsb.org/structure/6M9T, accessed on 21 December 2022) [42]. The PDB file was processed using Maestro (www.schrodinger.com, accessed on 21 December 2022) tools: Protein Preparation Wizard was used for refining the structure, to avoid clashes between atoms, and System Builder was used for calculating partial atomic charges using the OPLS3e force field [43]. Finally, the structure was converted to the mol2 format. Ligands PGE_2_ and L-798,106 were prepared using Maestro tool LigPrep applying an OPLS3e force field. Additionally, both molecules were processed with the tool LigPrep of Maestro to calculate the charges of each atom using the force field OPLS3e. The resulting structures were saved in the mol2 format.

When performing BD consensus runs, two docking engines, Autodock Vina [44] and Lead Finder [45], were selected, and a grid box size whose dimensions were 30 × 30 × 30 Å was specified with default parameters. All BD calculations were carried out using the MetaScreener suite of scripts (https://github.com/bio-hpc/metascreener, accessed on 21 December 2022). Lead Finder accepts protein and ligand files directly in the mol2 format, and for Vina, all the molecules were prepared using Gasteiger charges [46] with AutoDockTools (https://autodock.scripps.edu/, accessed on 21 December 2022) and converted to the pdbqt format. In the BD process, several docking calculations are performed in parallel, with one instance of the ligand starting on each alpha carbon of the protein model. Next, a distribution of docking score values as an approximation of binding energies and their structural clusters of poses was generated [41]. Finally, individual BD calculations obtained by each docking engine were discussed to obtain a BD consensus with the superposition and the scores of both methods. The scoring function of both algorithms considers the Lennard-Jones term (LJ), hydrogen bonds (H-bonds), electrostatic interactions, hydrophobic stabilization, entropic penalty due to the number of rotatable bonds, and the internal energy of each ligand. The interactions between the studied ligands and the residues of the protein were calculated using PLIP (https://plip-tool.biotec.tu-dresden.de/plip-web/plip/index, accessed on 21 December 2022).

### 4.9. Molecular Dynamics

For each ligand, the most relevant interactions cluster after BD calculations were selected and a specific ligand pose was specified. Next, such poses were selected to run a molecular dynamic simulation (MD), which allows to study the time evolution of the protein–ligand systems. In addition, and in order to compare with known experimental results, the crystallographic pose of misoprostol (PDB ligand code J9P) from the PDB entry 6M9T was set up with Maestro tools to run another MD. The three MD calculations were carried out with Maestro-Desmond software (Desmond Molecular Dynamics System, D. E. Shaw Research, New York, NY, 2020. Maestro-Desmond Interoperability Tools, Schrödinger, New York, NY, 2020). Protein–ligand complexes were inserted into a membrane of POPC (1-palmitoyl-2-oleoyl-sn-glycero-3-phosphocholine) [47] at 300 K, and the transmembrane region was obtained following the data of the UniProt database (https://www.uniprot.org/, accessed on 21 December 2022). The complexes created were immersed in a box filled with water molecules using the simple point charge (SPC) scheme. The dimension of the box was set to 10 × 10 × 10 Å. Cl^−^ and Na^+^ ions were added to obtain a final NaCl concentration of 0.15 M. Energy minimization was carried out by 2000 steps using the steepest descent method with a threshold of 1.0 kcal/mol/Å. Periodic boundary conditions were used, and a cutoff of 9 Å was established for van der Waals interactions, and the Particle Mesh Ewald (PME) method with a tolerance of 10^−9^ was used in the electrostatic part. The NPT simulations were realized at 300 K with the Nosé-Hoover algorithm [48], and the pressure was maintained at 1 bar with the Martyna–Tobias–Klein barostat [49]. The same force field used to prepare the molecules was used in all runs [43]. All the runs had a time of simulation of 100 ns.

### 4.10. Statistical Analysis

Continuous variables were tested for normality of distribution with the Kolmogorov–Smirnov test. Normal data are presented as mean standard deviation (SD) and non-normal data are presented as median (interquartile range). Comparisons were performed using analysis of variance (ANOVA) with Tukey’s post hoc analysis (normally distributed data) or Mann–Whitney U test (non-normally distributed data). The analyses were performed using SPSS 22.0 for Windows software (SPSS Inc., Chicago, IL, USA). A two-tailed probability value of *p* < 0.05 was considered significant in all statistical analyses.

## 5. Conclusions

In conclusion, 9-D1t-PhytoP is the most abundant oxylipin in the macroalgae *Gracilaria longissima*, and it influences human platelet activation in blood with/without ADP agonist stimulus. Furthermore, our results encourage future studies using 9-D1t-PhytoP instead of PGE2 or Sulprostone, because of its pro-aggregatory potential. This work estimated the critical interactions with the binding site residues of the EP3 receptor to decipher the biological activity reported here. The selective agonists of the platelet EP3 receptor such as 9-D1t-PhytoP and Misoprostol deserve further investigation as targeting newly designed compounds might develop novel clinical strategies.

## Figures and Tables

**Table 1:** Levels of oxylipins (PhytoPs and PhytoFs, ng/sample) in raw material, after methanol extraction.

	PhytoPs ^Z^									PhytoFs ^Y^		
Sample	FP-1	FP-2	FP-3	FP-4	FP-5	FP-6	FP-7	**Total**	FF-1	FF-2	FF-3	**Total**
1	38.5	**1591**	55.1	0.2	3.1	9.0	13.6	**1717.9**	171.9	23.1	13.6	**208.7**
2	25.2	**752**	26.7	<0.1	1.1	3.6	7.0	**815.7**	298.2	31.7	6.7	**336.6**
3	52.8	**1967**	44.8	0.2	3.4	10.0	12.1	**2100.6**	307.6	7.7	12.1	**326.8**

^Z^ FP-1, 9-F_1t_-PhytoP; **FP-2, 9-D_1t_-PhytoP**; FP-3, ent-16-F_1t_-PhytoP + *ent*-16-*epi*-16-F_1t_-PhytoP; FP-4, 16-B_1t_-PhytoP; FP-5, 9-*epi*-9-F_1t_-PhytoP; FP-6, 9-L_1t_-PhytoP; FP-7, 9-*epi*-9-D_1t_-PhytoP. ^Y^ FF-1, *ent*-16-(*RS*)-9-*epi*-ST-Δ^14^-10-PhytoF; FF-2, *ent*-16-(*RS*)-13-*epi*-ST-Δ^14^-9-PhytoF; FF-3, *ent*-9-(*RS*)-12-*epi*-ST-Δ^10^-13-PhytoF.

## Data Availability

Not applicable.

## Supplementary Materials

The following supporting information can be downloaded at: https://www.mdpi.com/article/10.3390/ijms24032730/s1.

## References

[1] Medina S., Gil-Izquierdo A., Durand T., Ferreres F., Dominguez-Perles R. (2018). Structural/Functional Matches and Divergences of Phytoprostanes and Phytofurans with Bioactive Human Oxylipins. Antioxidants.

[2] Collado-Gonzalez J., Cano-Lamadrid M., Perez-Lopez D., Carbonell-Barrachina A.A., Centeno A., Medina S., Grinan I., Guy A., Galano J.M., Durand T. (2020). Effects of Deficit Irrigation, Rootstock, and Roasting on the Contents of Fatty Acids, Phytoprostanes, and Phytofurans in Pistachio Kernels. J. Agric. Food Chem..

[3] Collado-Gonzalez J., Medina S., Durand T., Guy A., Galano J.M., Torrecillas A., Ferreres F., Gil-Izquierdo A. (2015). New UHPLC-QqQ-MS/MS method for quantitative and qualitative determination of free phytoprostanes in foodstuffs of commercial olive and sunflower oils. Food Chem..

[4] Medina S., Collado-Gonzalez J., Ferreres F., Londono-Londono J., Jimenez-Cartagena C., Guy A., Durand T., Galano J.M., Gil-Izquierdo A. (2017). Quantification of phytoprostanes—bioactive oxylipins—and phenolic compounds of Passiflora edulis Sims shell using UHPLC-QqQ-MS/MS and LC-IT-DAD-MS/MS. Food Chem..

[5] Pinciroli M., Dominguez-Perles R., Abellan A., Guy A., Durand T., Oger C., Galano J.M., Ferreres F., Gil-Izquierdo A. (2017). Comparative Study of the Phytoprostane and Phytofuran Content of indica and japonica Rice (Oryza sativa L.) Flours. J. Agric. Food Chem..

[6] Pinciroli M., Dominguez-Perles R., Garbi M., Abellan A., Oger C., Durand T., Galano J.M., Ferreres F., Gil-Izquierdo A. (2018). Impact of Salicylic Acid Content and Growing Environment on Phytoprostane and Phytofuran (Stress Biomarkers) in *Oryza sativa* L.. J. Agric. Food Chem..

[7] Lipan L., Collado-Gonzalez J., Dominguez-Perles R., Corell M., Bultel-Ponce V., Galano J.M., Durand T., Medina S., Gil-Izquierdo A., Carbonell-Barrachina A. (2020). Phytoprostanes and Phytofurans-Oxidative Stress and Bioactive Compounds-in Almonds are Affected by Deficit Irrigation in Almond Trees. J. Agric. Food Chem..

[8] Dominguez-Perles R., Abellan A., Leon D., Ferreres F., Guy A., Oger C., Galano J.M., Durand T., Gil-Izquierdo A. (2018). Sorting out the phytoprostane and phytofuran profile in vegetable oils. Food Res. Int..

[9] Carrasco-Del Amor A.M., Aguayo E., Collado-Gonzalez J., Guy A., Galano J.M., Durand T., Gil-Izquierdo A. (2016). Impact of packaging atmosphere, storage and processing conditions on the generation of phytoprostanes as quality processing compounds in almond kernels. Food Chem..

[10] Carrasco-Del Amor A.M., Aguayo E., Collado-Gonzalez J., Guy A., Galano J.M., Durand T., Gil-Izquierdo A. (2017). Impact of processing conditions on the phytoprostanes profile of three types of nut kernels. Free Radic. Res..

[11] Martinez Sanchez S., Dominguez-Perles R., Montoro-Garcia S., Gabaldon J.A., Guy A., Durand T., Oger C., Ferreres F., Gil-Izquierdo A. (2020). Bioavailable phytoprostanes and phytofurans from Gracilaria longissima have anti-inflammatory effects in endothelial cells. Food Funct..

[12] Campillo M., Medina S., Fanti F., Gallego-Gomez J.I., Simonelli-Munoz A., Bultel-Ponce V., Durand T., Galano J.M., Tomas-Barberan F.A., Gil-Izquierdo A. (2021). Phytoprostanes and phytofurans modulate COX-2-linked inflammation markers in LPS-stimulated THP-1 monocytes by lipidomics workflow. Free Radic. Biol. Med..

[13] Lara-Guzman O.J., Gil-Izquierdo A., Medina S., Osorio E., Alvarez-Quintero R., Zuluaga N., Oger C., Galano J.M., Durand T., Munoz-Durango K. (2018). Oxidized LDL triggers changes in oxidative stress and inflammatory biomarkers in human macrophages. Redox Biol..

[14] Markovic T., Jakopin Z., Dolenc M.S., Mlinaric-Rascan I. (2017). Structural features of subtype-selective EP receptor modulators. Drug Discov. Today.

[15] Petrucci G., De Cristofaro R., Rutella S., Ranelletti F.O., Pocaterra D., Lancellotti S., Habib A., Patrono C., Rocca B. (2011). Prostaglandin E2 differentially modulates human platelet function through the prostanoid EP2 and EP3 receptors. J. Pharmacol. Exp. Ther..

[16] Paul B.Z., Ashby B., Sheth S.B. (1998). Distribution of prostaglandin IP and EP receptor subtypes and isoforms in platelets and human umbilical artery smooth muscle cells. Br. J. Haematol..

[17] Iyu D., Juttner M., Glenn J.R., White A.E., Johnson A.J., Fox S.C., Heptinstall S. (2011). PGE1 and PGE2 modify platelet function through different prostanoid receptors. Prostaglandins Other Lipid Mediat..

[18] Philipose S., Konya V., Sreckovic I., Marsche G., Lippe I.T., Peskar B.A., Heinemann A., Schuligoi R. (2010). The prostaglandin E2 receptor EP4 is expressed by human platelets and potently inhibits platelet aggregation and thrombus formation. Arterioscler. Thromb. Vasc. Biol..

[19] Matthews J.S., Jones R.L. (1993). Potentiation of aggregation and inhibition of adenylate cyclase in human platelets by prostaglandin E analogues. Br. J. Pharmacol..

[20] Heptinstall S., Espinosa D.I., Manolopoulos P., Glenn J.R., White A.E., Johnson A., Dovlatova N., Fox S.C., May J.A., Hermann D. (2008). DG-041 inhibits the EP3 prostanoid receptor--a new target for inhibition of platelet function in atherothrombotic disease. Platelets.

[21] Kuriyama S., Kashiwagi H., Yuhki K., Kojima F., Yamada T., Fujino T., Hara A., Takayama K., Maruyama T., Yoshida A. (2010). Selective activation of the prostaglandin E2 receptor subtype EP2 or EP4 leads to inhibition of platelet aggregation. Thromb. Haemost..

[22] Schober L.J., Khandoga A.L., Dwivedi S., Penz S.M., Maruyama T., Brandl R., Siess W. (2011). The role of PGE(2) in human atherosclerotic plaque on platelet EP(3) and EP(4) receptor activation and platelet function in whole blood. J. Thromb. Thrombolysis.

[23] Schober L.J., Khandoga A.L., Penz S.M., Siess W. (2010). The EP3-agonist sulprostone, but not prostaglandin E2 potentiates platelet aggregation in human blood. Thromb. Haemost..

[24] Rinder C.S., Student L.A., Bonan J.L., Rinder H.M., Smith B.R. (1993). Aspirin does not inhibit adenosine diphosphate-induced platelet alpha-granule release. Blood.

[25] Lagarde M., Chen P., Vericel E., Guichardant M. (2010). Fatty acid-derived lipid mediators and blood platelet aggregation. Prostaglandins Leukot. Essent. Fatty Acids.

[26] Jadhav V., Jabre A., Lin S.Z., Lee T.J. (2004). EP1- and EP3-receptors mediate prostaglandin E2-induced constriction of porcine large cerebral arteries. J. Cereb. Blood Flow Metab..

[27] Shantsila E., Montoro-Garcia S., Lip G.Y. (2014). Monocytes circulate in constant reversible interaction with platelets in a [Ca2+]-dependent manner. Platelets.

[28] Montoro-Garcia S., Shantsila E., Hernandez-Romero D., Jover E., Valdes M., Marin F., Lip G.Y. (2014). Small-size platelet microparticles trigger platelet and monocyte functionality and modulate thrombogenesis via P-selectin. Br. J. Haematol..

[29] Rao R., Redha R., Macias-Perez I., Su Y., Hao C., Zent R., Breyer M.D., Pozzi A. (2007). Prostaglandin E2-EP4 receptor promotes endothelial cell migration via ERK activation and angiogenesis in vivo. J. Biol. Chem..

[30] Lau S., Gossen M., Lendlein A., Jung F. (2021). Venous and Arterial Endothelial Cells from Human Umbilical Cords: Potential Cell Sources for Cardiovascular Research. Int. J. Mol. Sci..

[31] Geenen I.L., Molin D.G., van den Akker N.M., Jeukens F., Spronk H.M., Schurink G.W., Post M.J. (2015). Endothelial cells (ECs) for vascular tissue engineering: Venous ECs are less thrombogenic than arterial ECs. J. Tissue Eng. Regen. Med..

[32] Martinez-Sanchez S.M., Perez-Sanchez H., Antonio Gabaldon J., Abellan-Aleman J., Montoro-Garcia S. (2019). Multifunctional Peptides from Spanish Dry-Cured Pork Ham: Endothelial Responses and Molecular Modeling Studies. Int. J. Mol. Sci..

[33] Hollenstein K. (2019). Structures shed light on prostanoid signaling. Nat. Chem. Biol..

[34] Morimoto K., Suno R., Hotta Y., Yamashita K., Hirata K., Yamamoto M., Narumiya S., Iwata S., Kobayashi T. (2019). Crystal structure of the endogenous agonist-bound prostanoid receptor EP3. Nat. Chem. Biol..

[35] Vigor C., Balas L., Guy A., Bultel-Poncé V., Reversat G., Galano J.M., Durand T., Oger C. (2022). Isoprostanoids, Isofuranoids and Isoketals—From Synthesis to Lipidomics. Eur. J. Org. Chem..

[36] van Velzen J.F., Laros-van Gorkom B.A., Pop G.A., van Heerde W.L. (2012). Multicolor flow cytometry for evaluation of platelet surface antigens and activation markers. Thromb. Res..

[37] Hechler B., Cattaneo M., Gachet C. (2005). The P2 receptors in platelet function. Semin. Thromb. Hemost..

[38] Alburquerque-Gonzalez B., Bernabe-Garcia A., Bernabe-Garcia M., Ruiz-Sanz J., Lopez-Calderon F.F., Gonnelli L., Banci L., Pena-Garcia J., Luque I., Nicolas F.J. (2021). The FDA-Approved Antiviral Raltegravir Inhibits Fascin1-Dependent Invasion of Colorectal Tumor Cells In Vitro and In Vivo. Cancers.

[39] Alburquerque-Gonzalez B., Bernabe-Garcia M., Montoro-Garcia S., Bernabe-Garcia A., Rodrigues P.C., Ruiz Sanz J., Lopez-Calderon F.F., Luque I., Nicolas F.J., Cayuela M.L. (2020). New role of the antidepressant imipramine as a Fascin1 inhibitor in colorectal cancer cells. Exp. Mol. Med..

[40] Livak K.J., Schmittgen T.D. (2001). Analysis of relative gene expression data using real-time quantitative PCR and the 2(-Delta Delta C(T)) Method. Methods.

[41] Tapia-Abellan A., Angosto-Bazarra D., Martinez-Banaclocha H., de Torre-Minguela C., Ceron-Carrasco J.P., Perez-Sanchez H., Arostegui J.I., Pelegrin P. (2019). MCC950 closes the active conformation of NLRP3 to an inactive state. Nat. Chem. Biol..

[42] Berman H.M., Westbrook J., Feng Z., Gilliland G., Bhat T.N., Weissig H., Shindyalov I.N., Bourne P.E. (2000). The Protein Data Bank. Nucleic Acids Res..

[43] Roos K., Wu C., Damm W., Reboul M., Stevenson J.M., Lu C., Dahlgren M.K., Mondal S., Chen W., Wang L. (2019). OPLS3e: Extending Force Field Coverage for Drug-Like Small Molecules. J. Chem. Theory Comput..

[44] Trott O., Olson A.J. (2010). AutoDock Vina: Improving the speed and accuracy of docking with a new scoring function, efficient optimization, and multithreading. J. Comput. Chem..

[45] Stroganov O.V., Novikov F.N., Stroylov V.S., Kulkov V., Chilov G.G. (2008). Lead finder: An approach to improve accuracy of protein-ligand docking, binding energy estimation, and virtual screening. J. Chem. Inf. Model.

[46] Gasteiger J., Marsili M. (1980). Iterative partial equalization of orbital electronegativity—A rapid access to atomic charges. Tetrahedron.

[47] Koynova R., Caffrey M. (1995). Phases and phase transitions of the sphingolipids. Biochim. Biophys. Acta.

[48] Tapias D., Sanders D.P., Bravetti A. (2016). Geometric integrator for simulations in the canonical ensemble. J. Chem. Phys..

[49] Martyna G.J., Tobias D.J., Klein M.L. (1994). Constant pressure molecular dynamics algorithms. J. Chem. Phys..

