# Supplementary material for: A Phytoprostane from Gracilaria longissima Increases Platelet Activation, Platelet Adhesion to Leukocytes and Endothelial Cell Migration by Potential Binding to EP3 Prostaglandin Receptor
Source: Int J Mol Sci. 2023 Feb 1;24(3):2730. doi: 10.3390/ijms24032730 (PMC9916792; doi:10.3390/ijms24032730)
Supplement: Supplementary file 1 [file ijms-24-02730-s001.zip › ijms-2168534-supplementary.pdf]

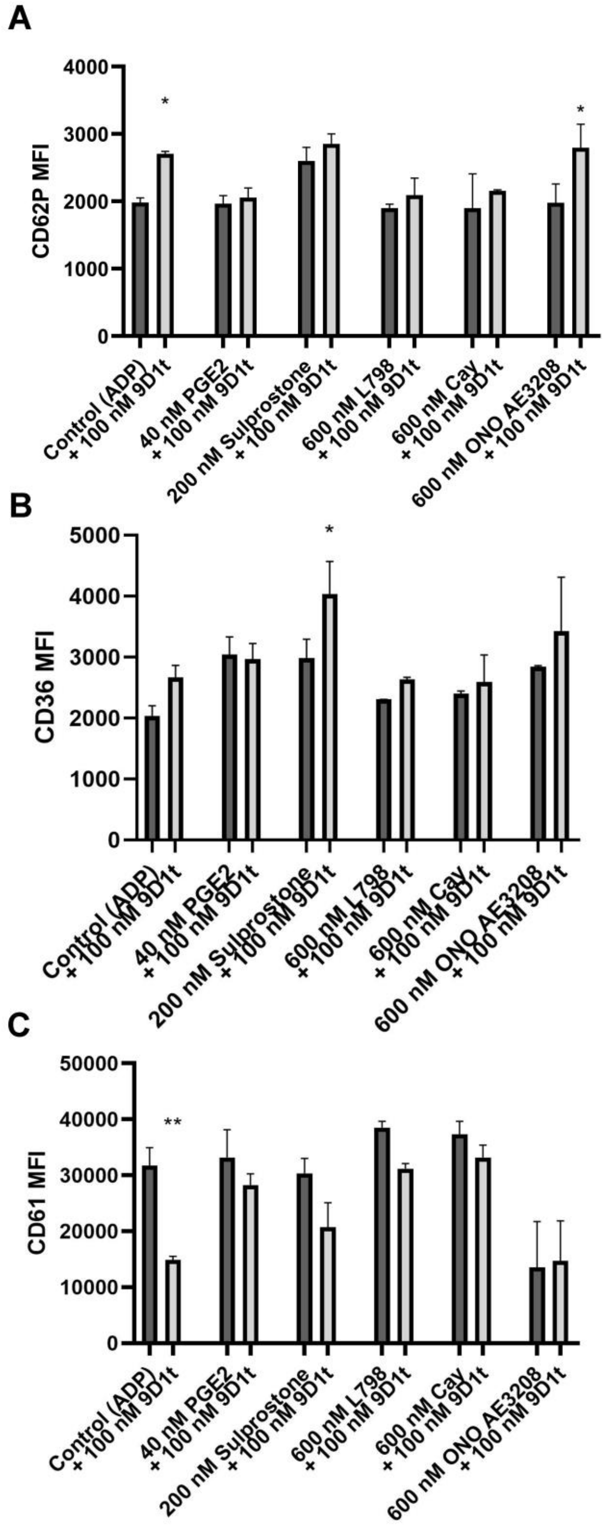

Figure S1. Effects of 9-D1t-PhytoP and EP receptor ligands on platelet activation markers, under ADP stimulation. (**A**) CD62P (P-selectin) expression. (**B**) CD36 (glycoprotein IV) expression. (**C**) CD61 (glycoprotein IIIa) expression. The final concentration of ADP in citrated whole blood was 20 µmol/L, for 2 min. MFI: mean fluorescence intensity. The bars show mean and standard deviation (n = 3 different blood donors). Stars denote significant differences as compared to samples without activation (control, ADP) */** = $p < 0.05/0.01$.

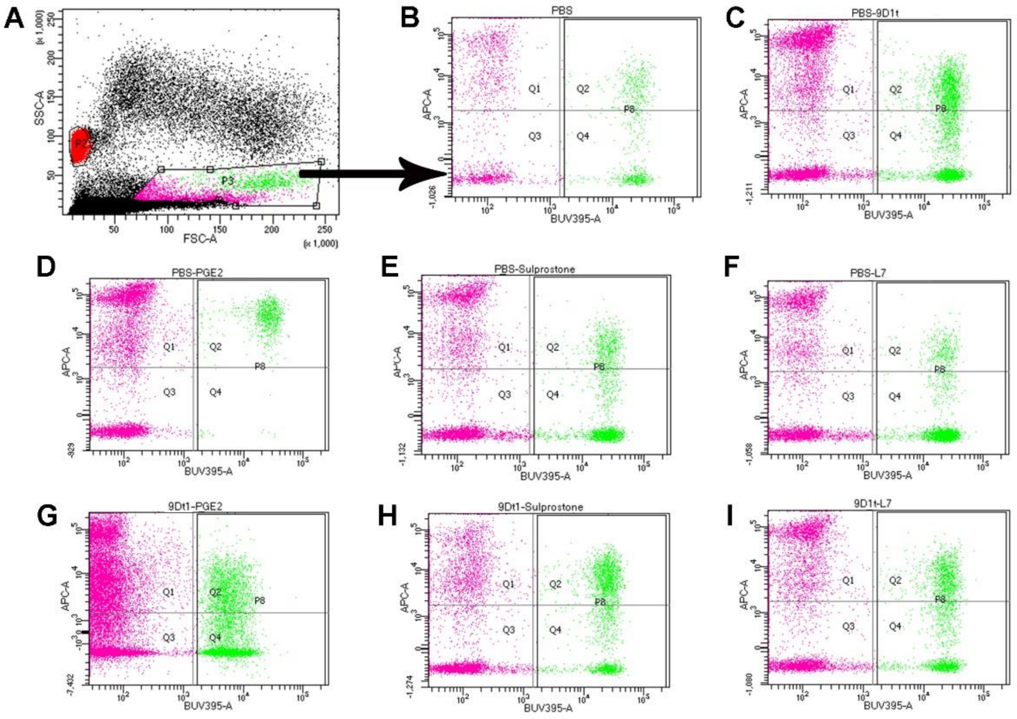

Figure S2. Gating strategy for monocytes and other PMNs cells (based on FSC and SSC). (**A**) P3 gate was further analyzed based on CD14-BUV395 (P8 gate) and CD42b-APC bindings (Q2 gate) in (**B**) control (PBS) conditions. After 30 min in the presence of: (**C**) 100 nM 9-D1t-PhytoP. (**D**) 20 nM PGE_2. (**E**) 200 nM Sulprostone. (**F**) 300 nM L798,106. (**G**) 20 nM PGE_2 plus 100 nM 9-D1t-PhytoP. (**H**) 200 nM Sulprostone plus 100 nM 9-D1t-PhytoP. (**I**) 300 nM L798,106 plus 100 nM 9-D1t-PhytoP.

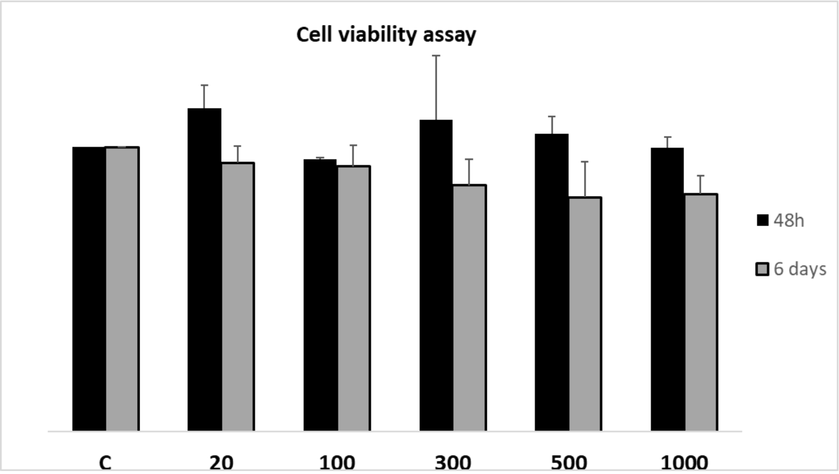

Figure S3. Cell viability assay performed with AlamarBLue reagent. Growing concentrations of 9-D1t-PhytoP up to 1 µg/mL were tested up to 6 days.

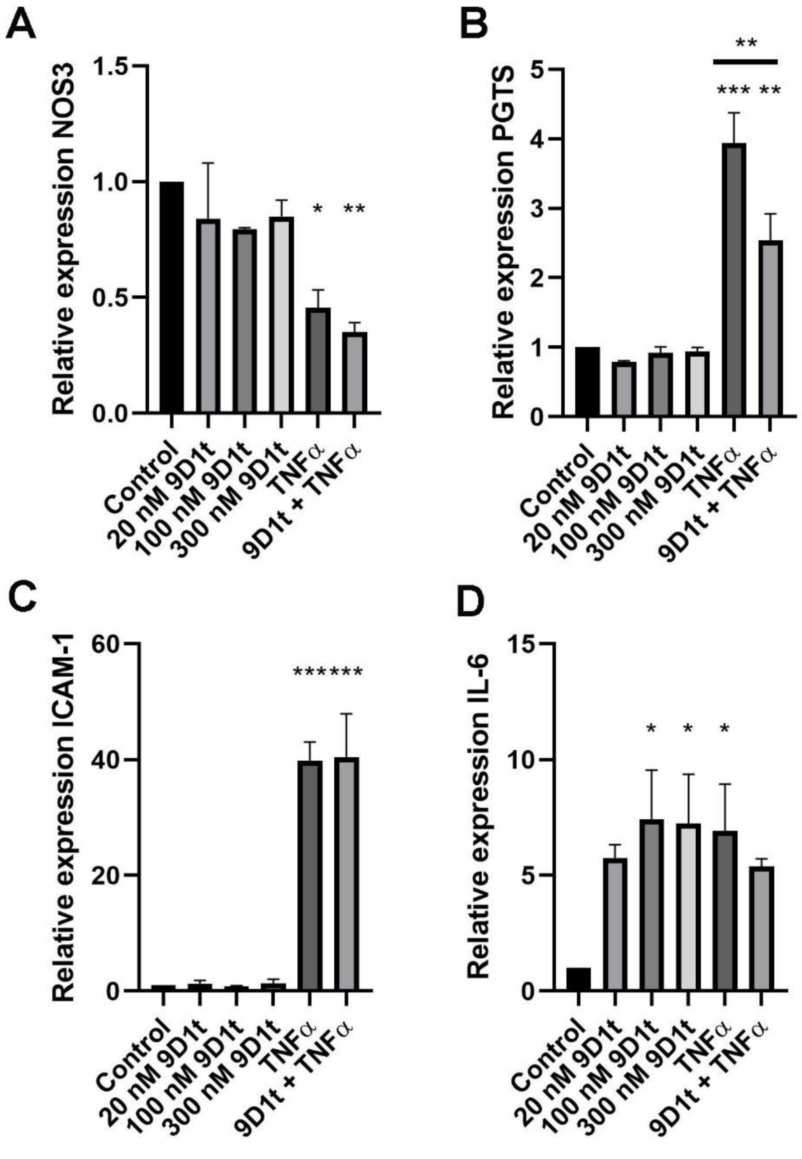

Figure S4. Relative mRNA expression in Ea.hy926 cells in the presence of 9-D1t-PhytoP. (**A**) NOS3; (**B**) PGST (COX-2); (**C**) ICAM-1; (**D**) IL-6, after treatment with 300 nM 9-D1t-PhytoP. Data shown represent averaged values of three independent experiments. Statistic differences compared with control conditions */**/*** = $p < 0.05/0.01/0.001$, respectively.

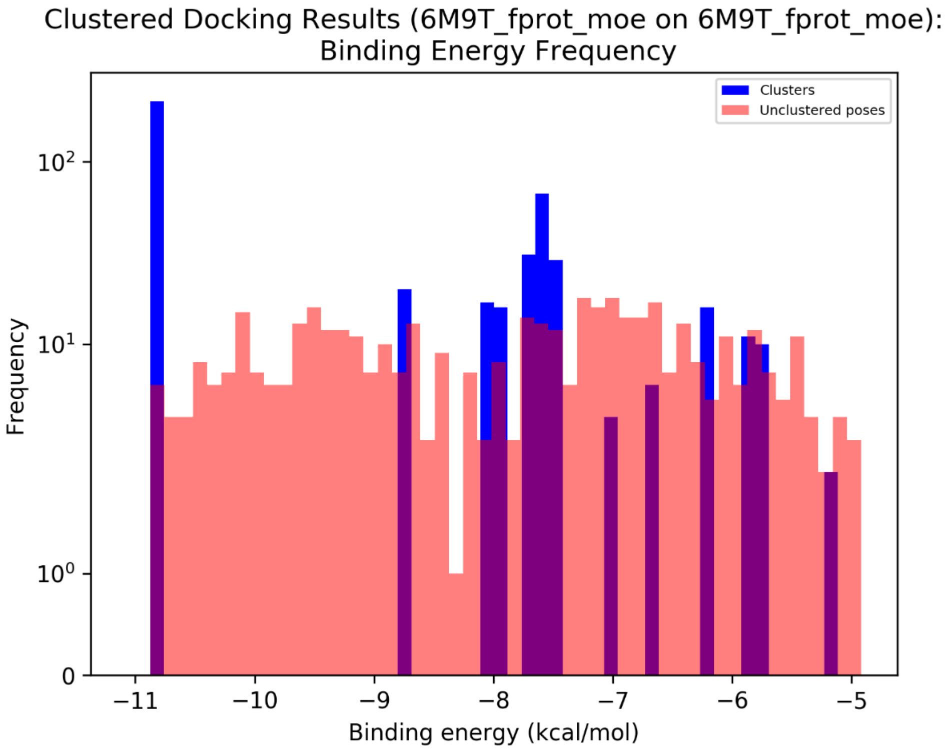

Figure S5. Interaction energy between 9D1t and the different clusters of the EP3 receptor.

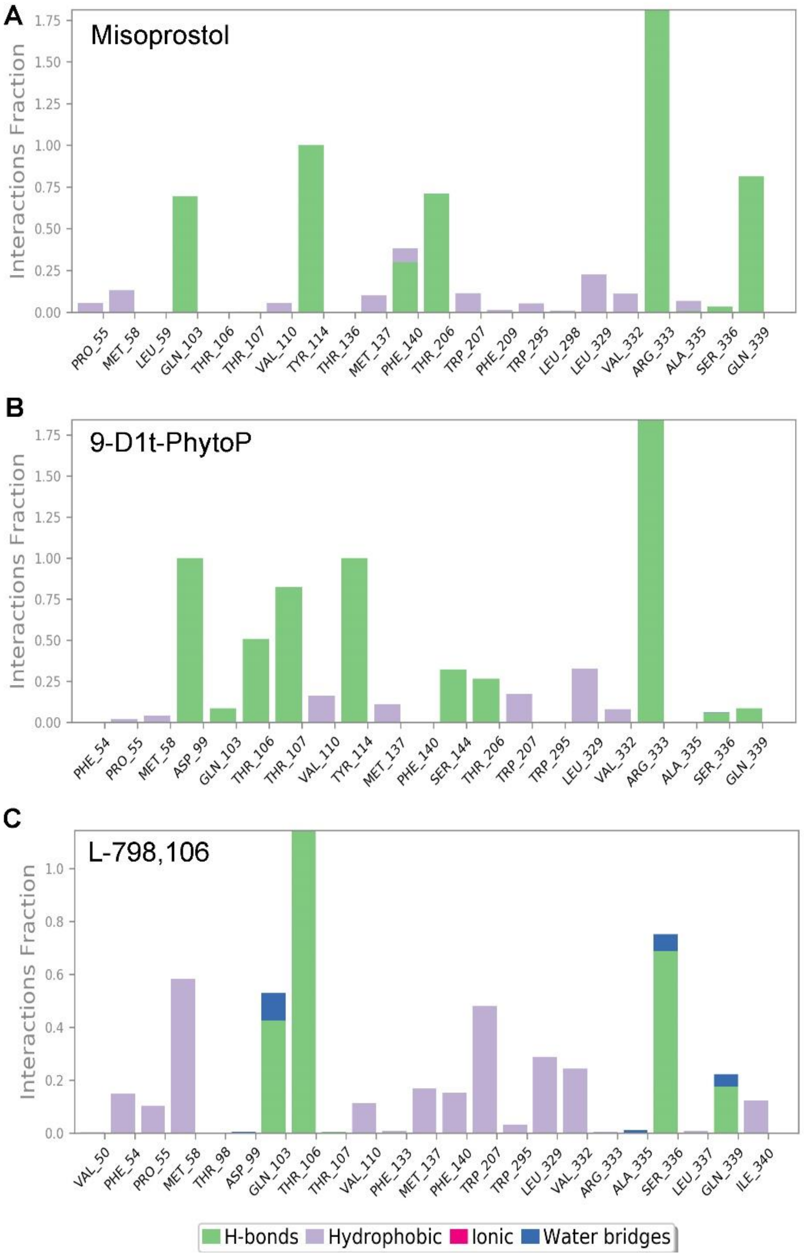

Figure S6. MD simulation, receptor–ligand interactions inside the binding pocket 100th nanosecond. The residue-wise binding energy comparison of (**A**) misoprostol; (**B**) 9-D1t-PhytoP and (**C**) L798,106.
